# Primary esophageal marginal zone lymphoma with respiratory symptoms: a case report and review of the literature

**DOI:** 10.3389/fmed.2026.1801520

**Published:** 2026-03-18

**Authors:** Xiaoyun Cheng, Wensi Zhang, Minggang Zhang, Shiyu Du

**Affiliations:** 1 Department of Gastroenterology, China Japan Friendship Hospital, Beijing, China; 2 Department of Gastroenterology, Peking University China-Japan Friendship School of Clinical Medicine, Beijing, China

**Keywords:** endoscopic ultrasound-guided fine needle aspiration (EUS-FNA), endoscopic examination, esophagus, marginal zone lymphoma, non-Hodgkin lymphoma

## Abstract

**Background:**

Primary esophageal lymphoma is a rare disease whose pathogenesis may be related to immune abnormalities. We report a case of primary esophageal marginal zone lymphoma (MZL) in the esophagus for 2 years, which initially presented with respiratory symptoms and was subsequently diagnosed by endoscopic ultrasound-guided fine-needle aspiration.

**Case presentation:**

A 68-year-old male presented with a 10-day history of cough, excessive sputum production, and wheezing after physical activity. Two years prior to this admission, the patient had sought medical attention at our hospital for fever and cough. After completing the relevant examinations, the preliminary diagnosis was considered to be a pulmonary infection, with the possibility of an esophageal neoplastic lesion. Upon readmission, a follow-up chest CT revealed thickening of the esophageal wall in multiple segments, which was more pronounced than it had been 2 years previously, and tracheal compression. Upper gastrointestinal endoscopy revealed a widespread, protruding lesion along the entire esophagus, with a smooth mucosal surface and no ulceration. Endoscopic ultrasound revealed the disappearance of the normal layered structure of the esophageal wall at the site of the lesion. PET-CT demonstrated uneven increased radioactive uptake throughout the esophagus. Bone marrow aspiration did not reveal any tumor involvement. Histopathological and immunohistochemical examinations revealed marginal zone lymphoma.

**Conclusion:**

Primary esophageal lymphoma has significant clinical heterogeneity, typically starting with progressive dysphagia and weight loss. This patient has a long history of atypical symptoms, representing the first case to present with respiratory symptoms without dysphagia. Due to the extremely low incidence of primary esophageal lymphoma and the lack of typical symptoms, imaging findings, and endoscopic manifestations, it is prone to misdiagnosis. Differential diagnosis is necessary to distinguish it from esophageal malignancies such as esophageal squamous cell carcinoma and esophageal adenocarcinoma, as well as benign esophageal conditions like esophagitis and esophageal tuberculosis. Performing endoscopic ultrasound-guided fine needle aspiration to complete pathological biopsy is crucial to reduce the risk of misdiagnosis and facilitate early diagnosis. Currently, after two courses of the G-CVP regimen (Obinutuzumab + Cyclophosphamide + Vincristine + Prednisone) demonstrated suboptimal efficacy, the treatment was switched to a triple combination of Obinutuzumab, Orelabrutinib, and Lenalidomide, which has now completed six courses.

## Introduction

Primary esophageal lymphoma is extremely rare, accounting for less than 1% of all gastrointestinal lymphomas (1). To date, fewer than 30 cases of primary esophageal lymphoma have been reported worldwide (2). The exact etiology remains unclear, but immunosuppression is considered an important risk factor, with a higher incidence observed in immunocompromised individuals (such as HIV-infected patients) (3–5). According to the current literature, clinical manifestations of primary esophageal lymphoma typically include progressive dysphagia and weight loss (6). Diagnosis is primarily based on endoscopic examination, biopsy, and immunohistochemical analysis. Treatment generally includes chemotherapy, often in combination with immunotherapy, radiation therapy, or surgery, depending on the pathological subtype and individual patient factors.

## Case presentation

A 68-year-old male presented with a 10-day history of cough, excessive sputum production, and wheezing after physical activity, which developed without an obvious trigger. He reported no dysphagia or significant weight change. Physical examination was unremarkable, with no palpable lymphadenopathy or hepatosplenomegaly. Two years prior to this admission, the patient had sought medical attention at our hospital for fever and cough. A chest CT at that time revealed viral pneumonia and an irregular thickening of the esophagus, raising suspicion of a tumor. As the patient reported no other discomfort and did not pay close attention to these residual abnormalities, no further investigations were pursued at that time. In this context, the differential diagnosis mainly includes esophageal malignancies such as esophageal squamous cell carcinoma and esophageal adenocarcinoma, as well as benign esophageal conditions such as esophagitis and esophageal tuberculosis.

Upon admission, laboratory tests revealed the following results: white blood cell count of 6.95 × 10^9^/L, red blood cell count of 2.76 × 10^12^/L, hemoglobin level of 101 g/L, platelet count of 409 × 10^9^/L, total lymphocyte count of 0.96 × 10^9^/L, and reticulocyte percentage of 3.55%. The immunoglobulin M level was elevated at 713 mg/dL, complement C4 level was 15.4 mg/dL, the C1q circulating complex level was 392 mg/L, and the serum cystatin C level was 1.14 mg/L. HIV testing was negative. Lactate dehydrogenase at 183 U/L and C-reactive protein at 2.86 mg/L were both within normal limits. However, the CD4^+^ T-cell count was 265 cells/μL, which is below the normal reference range, indicating an underlying immunosuppressive state in the patient.

**Figure 1:**
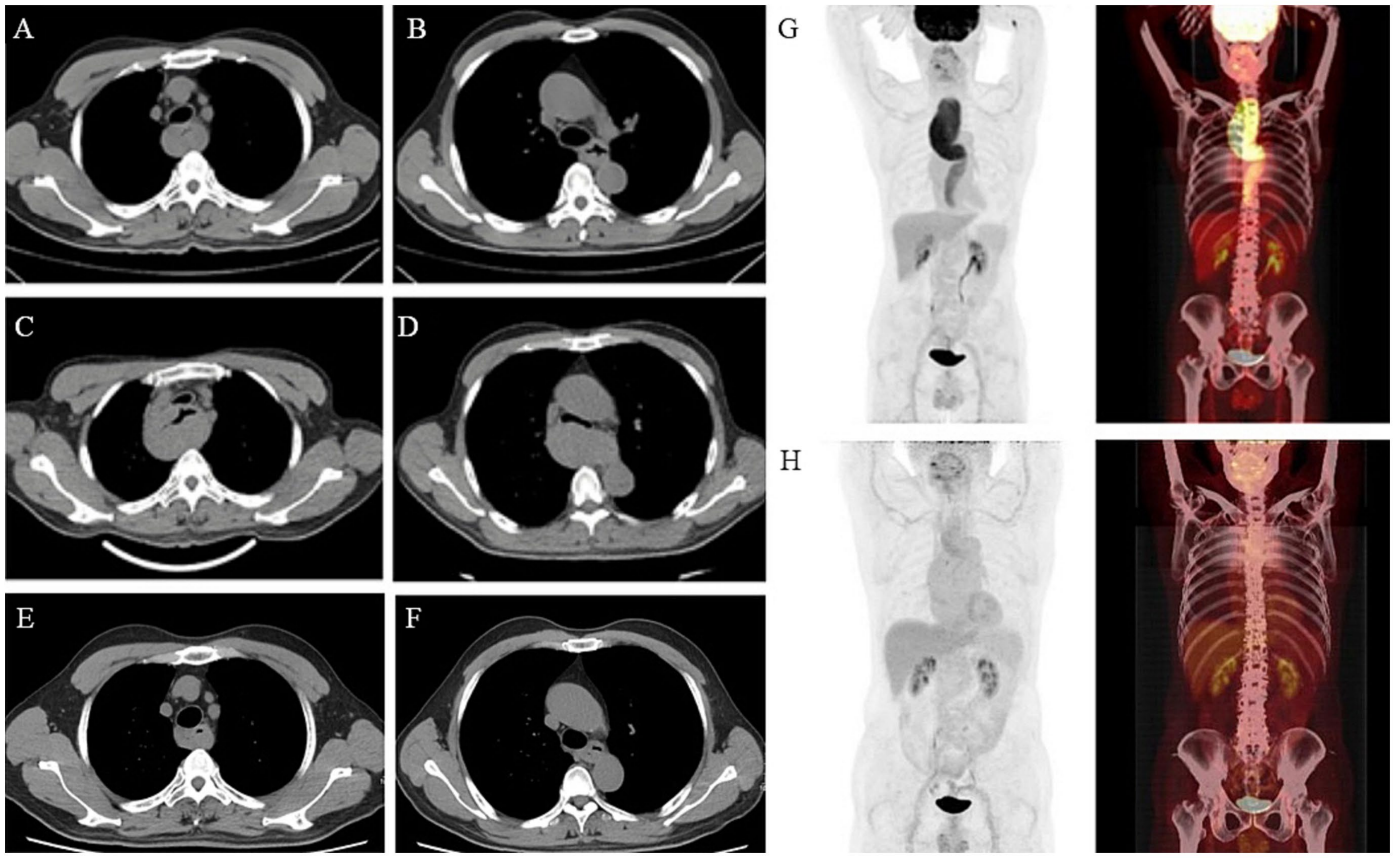
Findings of chest CT and PET-CT in 2022, 2024, and 2025. **(A,B)** Chest CT in 2022: multisegmental thickening of the esophageal wall and slight local compression of the trachea. **(C,D)** Chest CT taken in 2024 revealed that the esophageal mucosa thickened significantly with severe local pressure in the trachea and that there was no significant lymph node enlargement in the mediastinum. **(E,F)** Chest CT in 2025: significant improvement in esophageal mucosal thickening compared to previous scans, with reduced compression on the trachea. **(G)** PET-CT in 2024: revealed irregular thickening of the entire esophageal wall and an uneven increase in radioactivity uptake. **(H)** PET-CT in 2025: reduced esophageal wall thickening compared to previous scans, suggesting partial remission.

**Figure 2:**
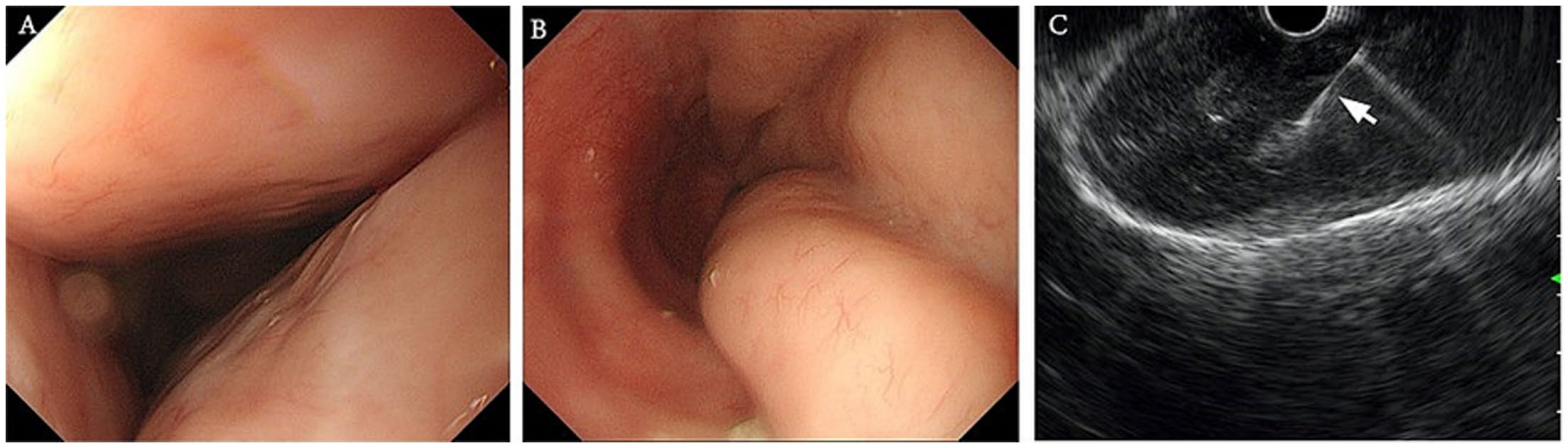
Findings of white light endoscopy and endoscopic ultrasound in 2024. **(A)** Esophagogastroduodenoscopy (EGD) in a forward endoscopic view revealed extensive uplifting lesions in the upper esophagus. **(B)** EGD revealed longitudinal uplifting lesions in the lower esophagus. **(C)** Endoscopic ultrasound demonstrated thickening of the esophageal wall, with the arrow indicating a hyperechoic puncture needle.

**Figure 3:**
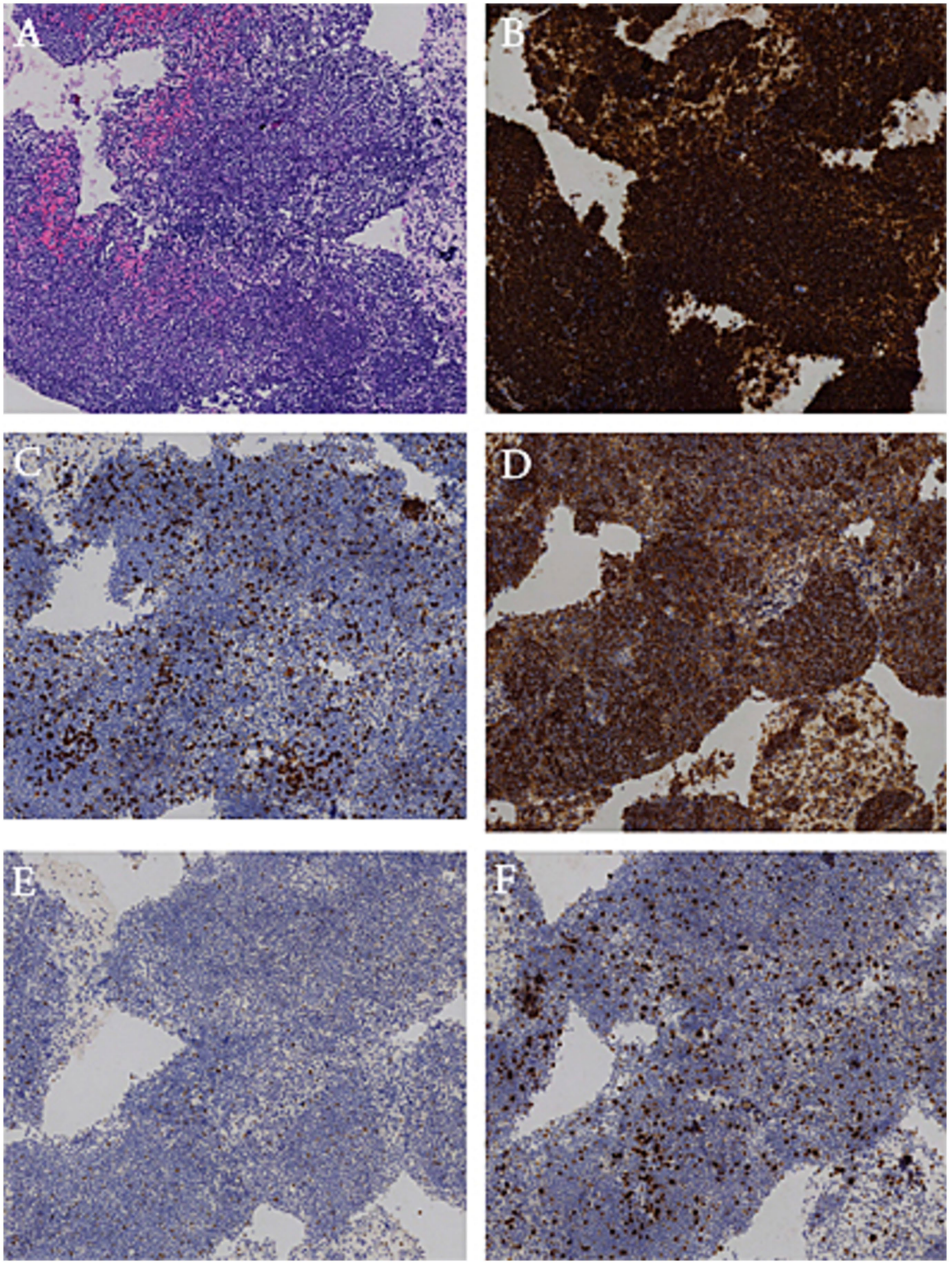
Histopathological analysis and immunohistochemical examination of the resected specimen. Diffuse infiltration of atypical lymphocytes in the marginal zone of lymphoid follicles. **(A)** HE. **(B)** CD20 (+). **(C)** CD3 (−). **(D)** CD21 (+). **(E)** Bcl6 (−). **(F)** Ki-67 (20%). Original magnification 100×.

Two years prior, the patient’s chest CT revealed multisegmental thickening of the esophageal wall with mild local compression of the trachea (Figures 1A,B). Upon readmission, a follow-up chest CT demonstrated significantly increased thickening of the esophageal mucosa compared with that 2 years prior, with severe local tracheal compression. No obvious enlargement of the mediastinal lymph nodes was observed (Figures 1C,D). To further assess the benign or malignant nature of the lesion, identify the primary site, and evaluate the extent of disease involvement, a PET-CT scan was performed. PET-CT revealed irregular thickening of the entire esophageal wall, particularly in the upper chest segment, with uneven increased radioactive uptake (SUVmax: 8.9) and tracheal compression (Figure 1G). Esophagogastroduodenoscopy (EGD) revealed extensive uplifting lesions in the upper esophagus and longitudinal uplifting lesions in the lower esophagus, with the mucosal surface mostly smooth and intact, without ulcerative lesions. No abnormalities were found in the stomach or duodenum (Figures 2A,B). Endoscopic ultrasound (EUS) revealed the disappearance of the normal layered structure of the esophageal wall at the site of the lesion, with blood flow signals detected within the lesion (Figure 2C). The histopathological findings revealed that the lesion was indolent B-cell lymphoma, with the following immunohistochemical results: CD20(+), CD79a(+), CD3 (few +), CD5 (few +), Bcl6(−), CD10(−), Ki67 (MIB-1) (approximately 20%+), and EBER(−). The combination of histopathology and immunohistochemistry suggested a diagnosis of marginal zone lymphoma (Figure 3).

In the same year, the patient consulted the hematology department. Tests for tuberculosis, Epstein–Barr virus, cytomegalovirus, and hepatitis B virus were all negative. Bone marrow aspiration revealed hypoactive marrow proliferation with no significant evidence of tumor involvement. KRAS and NRAS gene mutation analyses were also negative, and no abnormalities were detected in the immunoglobulin gene rearrangement test. Immunohistochemical analysis revealed the following: CD10 (partial +), CD117 (−), CD138 (partial +), CD235a (erythroid +), CD3 (+), CD34 (vascular +), CD5 (+), CD61 (megakaryocytic +), CD79α (sporadic +), Cyclin D1 (−), MPO (myeloid +), TdT (−), and CD20 (L26) (scattered +). The findings were consistent with marginal zone lymphoma. The patient was diagnosed with primary esophageal marginal zone lymphoma (MZL) (Ann Arbor-Cotswold Stage IE).

On the basis of the PET–CT results, the lymphoma was considered to be primary and confined to the esophagus and was staged as stage 1E. Currently, after two courses of the G-CVP regimen (Obinutuzumab + Cyclophosphamide + Vincristine + Prednisone) demonstrated suboptimal efficacy, the treatment was switched to a triple combination of Obinutuzumab, Orelabrutinib, and Lenalidomide, which has now completed six courses. The patient’s cough and wheezing after exercise improved. After completion of treatment, follow-up chest CT showed that the esophageal mucosal thickening had significantly improved compared to previous scans, and the tracheal compression was alleviated (Figures 1E,F). Follow-up PET-CT revealed that the esophageal wall thickening was reduced compared to previous scans, and no suspicious new lesions were found, suggesting partial remission (Figure 1H). The patient is still being closely monitored.

## Discussion

Primary esophageal lymphoma is an extremely rare and unique clinical entity, accounting for less than 1% of all gastrointestinal lymphomas1. Most cases of primary esophageal MZL are linked to immunosuppressive conditions, such as HIV/AIDS, suggesting that immune dysfunction plays a significant role in its pathogenesis (3–5). Although this patient did not present with obvious immunodeficiency, immunological tests revealed elevated levels of immunoglobulin M, complement C4, and C1q. The exact underlying mechanism remains unclear, and further research is needed to better understand the genetic and molecular factors contributing to its development.

Clinically, primary esophageal MZL presents with nonspecific symptoms such as dysphagia and weight loss (6). The clinical manifestations are highly heterogeneous, and diagnosis relies heavily on pathological results from biopsy due to the absence of specific diagnostic tests and endoscopic or imaging findings. These nonspecific features often lead to misdiagnosis as other esophageal diseases, such as squamous cell carcinoma, adenocarcinoma, esophagitis, leiomyoma, or tuberculosis, which may present similarly on imaging as wall thickening or submucosal masses. This diagnostic challenge highlights the main focus of this case report: not the treatment itself, but the difficulty in detecting primary esophageal MZL due to its rarity and lack of distinctive features. Therefore, even in cases where imaging only shows esophageal wall thickening and the patient presents with mild symptoms, clinicians should include primary esophageal lymphoma in the differential diagnosis to raise awareness of this disease and avoid missed diagnosis and misdiagnosis. With advancements in endoscopic techniques, this patient underwent endoscopic biopsy through endoscopic ultrasound-guided fine-needle aspiration (EUS-FNA), avoiding the trauma associated with traditional surgical procedures. The treatment of MZL typically includes surgery, chemotherapy, and radiotherapy. However, owing to the limited number of cases, the lack of large-scale studies, and insufficient long-term follow-up data, treatment strategies and prognostic outcomes have not been fully standardized. A literature review up to March 1, 2026, identified 27 reported cases of primary esophageal MZL, 23 of which included treatment details (Table 1). Among these, 11 patients underwent surgical treatment (7–17), including 3 patients who underwent endoscopic resection (14–17). Three patients received adjuvant radiotherapy or chemotherapy following surgery (7, 11, 17). There were 3 patients treated with radiotherapy alone (18–20), 3 patients treated with chemotherapy alone (21–23), 1 patient treated with immunotherapy alone (24), and 6 patients treated with a combination of chemotherapy and immunotherapy (2, 6, 25–28). In the remaining 3 reports, only the diagnosis was mentioned without treatment details (29–31). Most patients achieved remission after treatment. However, 1 patient experienced relapse after radiotherapy (13), and 1 patient developed mediastinal lymph node granulomatous nodules after combined chemoimmunotherapy (27). Notably, with the advancement of endoscopic techniques, diagnosis increasingly relies on endoscopic procedures rather than surgical interventions. In the past 8 years, 4 newly reported cases were primarily diagnosed through endoscopy. Among them, 1 patient with mucosa-confined disease was completely resected by endoscopic submucosal dissection (ESD) (14), while the other 3 patients were diagnosed through endoscopic biopsy and subsequently treated with combined chemoimmunotherapy, all of which produced favorable responses (2, 6, 26). In accordance with the latest guidelines (32), this patient was treated with the G-CVP regimen (cyclophosphamide, vincristine, prednisone, and obinutuzumab) and has completed two cycles of therapy, but the treatment efficacy has been suboptimal. Currently, the triple combination regimen of Obinutuzumab + Orelabrutinib + Lenalidomide has not been listed as a standard recommended therapy in international mainstream guidelines. However, clinical trials are exploring its potential, and it is primarily used for aggressive or refractory B-cell non-Hodgkin lymphomas (NHL), especially as salvage therapy after failure of standard treatments.

**Table 1:** Summary of the reported cases of primary esophageal mucosa-associated lymphoid tissue lymphoma.

Ref	Age	Sex	Symptoms	Tumor			Treatment		Follow-up period (mo)	Outcome
				Location	Size, cm	Diagnostic method	Primary	Adjuvant		
2	62	F	Dysphagia and weight loss	U-L	2.7	Biopsy	Chemotherapy and immunotherapy		NA	CR
6	68	F	Dysphagia and weight loss	U-L	7	Biopsy	Chemotherapy and immunotherapy		NA	CR
7	66	F	Dysphagia	M and L	2.7 × 2.1 × 4.8 and 2.2 × 2.2 × 3.0	Surgery	Surgery	Chemotherapy	NA	CR
8	50	M	NA	M-L	10	Surgery	Surgery		12	CR
9	49	M	None	M	NA	Surgery	Surgery		12	CR
10	75	M	NA	U-L	14 × 3.5 × 2.5	Surgery	Surgery		8	CR
11	53	M	Dysphagia	M	3.5 × 2.5 × 0.7	Surgery	Surgery	Radiotherapy	8	CR
12	61	M	Dysphagia	U-L	2 × 8	Surgery	Surgery		1	CR
13	55	M	None	M	5	Surgery	Surgery		21	Relapse (stomach)
14	62	F	Dysphagia	U-L	15.0 × 6.0	ESD	Esd		12	CR
15	83	F	Heartburn	U	1	EMR	Emr		22	CR
16	77	F	Dysphagia	L	4.3 × 2.8 × 1.5	ESD	Esd		5	CR
17	70	F	None	M	0.6 × 0.4 and 2.0 × 0.8	Biopsy	Emr	Radiotherapy	36	CR
18	76	M	Muscular weakness	M	NA	Biopsy	Radiotherapy		12	CR
19	59	M	Tarry stool	U-L	15 × 6.5 × 6	Biopsy	Radiotherapy		36	CR
20	70	F	None	L	2	Biopsy	Radiotherapy		13	CR
21	65	M	Dysphagia	U	10 × 3 × 3	Biopsy	Chemotherapy		24	CR
22	49	M	Hematochezia	M	0.8	Biopsy	Chemotherapy		6	CR
23	61	M	Upper gastrointestinal bleeding	U	1.2	Biopsy	Chemotherapy		NA	NA
24	70	M	Dysphagia	M	5	Biopsy	Immunotherapy		6	PR
25	60	F	Dysphagia	U-L	NA	Biopsy	Chemotherapy and immunotherapy		NA	CR
26	85	M	Dysphagia	U-L	NA	Biopsy	Chemotherapy and immunotherapy		NA	CR
27	60	F	NA	L	5 × 2 × 0.5	Biopsy	Chemotherapy and immunotherapy		24	CR (granulomatous nodules)
28	72	F	Anemia	M	2	Biopsy	Chemotherapy and immunotherapy		12	PR
29	63	F	NA	U	10	Biopsy	Na		NA	NA
30	37	M	NA	NA	NA	Biopsy	Na		NA	NA
31	72	M	NA	NA	>10	Biopsy	Na		NA	NA
Present case	68	M	Cough, excessive sputum production and wheezing	U-L	>10	Biopsy	Chemotherapy and immunotherapy		12	PR

U, upper; M, middle; L, lower; EMR, endoscopic mucosal resection; ESD, endoscopic submucosal dissection; CR, complete remission; PR, partial remission; NA, not available.

## Conclusion

In conclusion, primary esophageal MZL is a rare but treatable type of lymphoma, and timely and thorough endoscopic biopsy is crucial, especially in immunocompromised patients. With appropriate treatment, the prognosis is generally favorable. However, further research is needed to better understand its pathogenesis and to optimize treatment strategies, aiming to improve patient outcomes and guide clinical decision-making.

## Data Availability

The original contributions presented in the study are included in the article/supplementary material, further inquiries can be directed to the corresponding author.
